# ﻿*Rubussemiplenus* (Rosaceae), a new species with naturally occurring semi-double flowers from Hunan, China

**DOI:** 10.3897/phytokeys.257.150519

**Published:** 2025-06-04

**Authors:** Ting-Ting Wang, Ming-Hong Li, Dai-Yong Kuang, Jiang-Lin Xia, Qiu-Ping Wang, Huan-Chong Wang

**Affiliations:** 1 School of Ecology and Environmental Science, Yunnan University, Kunming 650091, Yunnan, China Yunnan University Kunming China; 2 Hunan Hengshan National Nature Reserve Administration, Hengyang, 421900, Hunan, China Hunan Hengshan National Nature Reserve Administration Hengyang China; 3 Hunan Nanyue Arboretum, Hengyang, 421900, Hunan, China Hunan Nanyue Arboretum Hengyang China; 4 Herbarium of Yunnan University, Kunming 650091, Yunnan, China Herbarium of Yunnan University Kunming China

**Keywords:** Endemism, Hengshan Mountains, morphology, phylogeny, Section *Rosaefolii*, taxonomy

## Abstract

The semi-double (*semiplenus*) floral trait is highly valued in the field of horticulture, yet it remains exceptionally rare in wild plants. *Rubussemiplenus*, a new species with naturally occurring semi-double flowers, is described and illustrated. This new species was discovered in the Nanyue Hengshan National Nature Reserve, Hunan Province, China. It is distinguished by its herbaceous to dwarf subshrub habit, simple suborbicular leaves (0.7–2.5 × 0.7–2.5 cm), and semi-double flowers with dicyclic petals (10–12). Molecular phylogenetic analyses based on ITS and *rbc*L + *mat*K sequences consistently confirmed that *R.semiplenus* belongs to Sect. Rosaefolii as defined by Focke and is closed to *R.hirsutus*.

## ﻿Introduction

*Rubus* L. is one of the largest genera in the Rosaceae family, belonging to the tribe Rubeae of subfamily Rosoideae (Yu and Lu 1985; Lu and Boufford 2003; Potter et al. 2007). This genus is found on all continents except Antarctica, with its primary distribution in East Asia and North America (Thompson 1995, 1997), and contains approximately 750–1000 species worldwide (Yu and Lu 1985; Jennings 1988). *Rubus* is one of the most taxonomically challenging genera of angiosperms (Wang et al. 2016; Okada et al. 2020; Gao et al. 2023; Huang et al. 2023). Species within this genus are typically shrubs, subshrubs, or perennial creeping herbs. Their leaves can be either compound or simple, and flowers are generally bisexual, although unisexual flowers occur rarely; fruits are fleshy aggregates of drupelets (Yu and Lu 1985; Kalkman 1993; Naruhashi 2001; Lu and Boufford 2003). Plants of *Rubus* have significant value in terms of food, medicine, ecology, and economy (Chen and Wang 2006; Foster et al. 2019).

The latest global revision of the genus *Rubus* was published nearly a century ago by Focke (1910, 1911, 1914), in which he divided the genus into twelve subgenera. In this classification, the majority of species were placed in R.subg.Rubus (= *Eubatus*), R.subg.Malachobatus (Focke) Focke, and R.subg.Idaeobatus (Focke) Focke. Focke’s subgeneric classification has been widely accepted and applied (Nakai 1918; Bailey 1941–1945; Naruhashi 2001). However, modern molecular systematics have not fully supported Focke’s classification (Alice and Campbell 1999; Yang and Pak 2006; Yang et al. 2012; Wang et al. 2016). More recently, Huang et al. (2023) proposed a new subgeneric classification system for *Rubus*, based on molecular phylogenetic analysis and morphological data, which divides the genus into 10 subgenera.

China has a rich diversity of *Rubus*, particularly in the southwestern region (Lu 1983; Gu et al. 2000; Lu and Boufford 2003). The first comprehensive revision of the genus *Rubus* in China was conducted by Yu and Lu (1985) for the “Flora Reipublicae Popularis Sinicae”, in which 194 species were recognized and classified into 8 sections and 24 subsections. Later, Lu and Boufford (2003) reviewed the Chinese species in the “Flora of China”, recognizing 208 species in China, with 139 of them being endemic. In recent years, numerous new species have been continuously discovered and published in China (e.g. Wang and Wang 2019; Wang et al. 2019; Wang et al. 2022; Chen et al. 2024a, 2024b), highlighting the need for continued investigation and study of *Rubus* diversity in China.

During a recent botanical expedition in Nanyue Hengshan National Nature Reserve, Hunan Province, China, we discovered an unknown species of *Rubus* characterized by its herbaceous to dwarf subshrub habit, simple leaves and semi-double flowers. Intrigued by its unique characteristics, we undertook a thorough examination of its morphological traits and performed phylogenetic analyses. The results of these studies clearly supported that this plant is a previously undescribed species.

## ﻿Materials and methods

### ﻿Morphological analyses

Morphological investigations were conducted in accordance with standard protocols for plant taxonomic surveys and herbarium-based classification (Davis and Heywood 1963). Specimen dissection and morphological examinations were performed at the laboratory in Yunnan University. A comprehensive list of morphological features was analyzed, including leaf shape (length, width), petiole length, stipule length, flower structure (size, number of petals, stamen count), fruit dimensions, seed morphology, and other distinctive vegetative and reproductive structures. These features were studied under a stereomicroscope (Olympus SZX2, Tokyo, Japan), and measurements were made using a ruler or a micrometer. Digital images available at the JSTOR Global Plants (http://plants.jstor.org/) and the Chinese Virtual Herbarium (https://www.cvh.ac.cn/), as well as relevant collections housed at CDBI, KUN, PE, PYU and YUKU (acronyms according to Thiers 2022), were examined and compared with the new species. Pertinent taxonomic literature (e.g. Focke 1910, 1911, 1914; Yu and Lu 1985; Lu and Boufford 2003; Wang et al. 2016; Wang et al. 2019) was extensively consulted.

### ﻿Phylogenetic study

To determine the phylogenetic position of the new species, ITS and two plastid genes (*rbc*L and *mat*K) were used as molecular markers. Total genomic DNA was extracted from silica-gel dried leaves of this new species using the DNAsecure Plant Kit (TIANGEN, Beijing, China).

The ITS primers used were ITS4 and ITS5, as detailed by White et al. (1990), and the PCR protocols were conducted according to Wang et al. (2016). Bidirectional sequencing was performed using an ABI 3730xL DNA Analyzer (Applied Biosystems) at the Kunming Branch of Beijing Qingke Biotechnology Co., Ltd. (Yunnan, China).

The new species was sequenced using the genome skimming technique (Straub et al. 2012; Zeng et al. 2018). DNA library construction and paired-end sequencing were completed by Novogene Bioinformatics Technology Co., Ltd. (Tianjin, China), with sequencing performed on the Illumina NovaSeq 6000 platform, generating approximately 3 Gbp of data. The chloroplast genomes (plastomes) were assembled from the clean data using GetOrganelle (Jin et al. 2020), and annotated the plastomes using GeSeq (Plastid Genome Annotator) (Tillich et al. 2017), followed by manual verification in Geneious (Kearse et al. 2012). Finally, the *mat*k and *rbc*L gene sequences were extracted separately from the assembled complete chloroplast genome (GenBank: PV590107) using Geneious.

A total of 65 ITS sequence datasets were obtained in this study, including a new ITS sequence of *R.semiplenus*, 60 *Rubus* sequences and four outgroup sequences obtained from GenBank (Appendix 1: Table A1). In addition, 54 *rbc*L and *mat*K sequences were downloaded from GenBank (Appendix 1: Table A2). Sequences were aligned using MAFFT (Katoh and Standley 2013). The combined dataset of *rbc*L + *mat*K was generated using the Connected Sequence plugin in PhyloSuite (Zhang et al. 2020). The sequences and accession numbers used in this study are detailed in Appendix 1.

Phylogenetic analyses were performed using Maximum Likelihood (ML) methods. The gap sites were removed using trimAl (Capella-Gutiérrez et al. 2009). To determine the nucleotide substitution models for the dataset, we used ModelFinder (Kalyaanamoorthy et al. 2017) to evaluate multiple nucleotide substitution models based on Bayesian Information Criterion (BIC) and selected the best-fit model. ML analysis was then performed using IQ-tree (Nguyen et al. 2015) with 1000 bootstrap replicates. The resulting phylogenetic tree was visualized using iTOL (Letunic and Bork 2021).

## ﻿Taxonomy

### ﻿ Rubus semiplenus

Taxon classificationPlantaeRosalesRosaceae

Huan C. Wang, Ming Hong Li & T. T. Wang sp. nov.

64389E58-5BC8-5DF9-B7D5-F039D77ED660

urn:lsid:ipni.org:names:77362791-1

Figs 1 , 2

#### Type.

China • Hunan Province: Nanyue Hengshan National Nature Reserve, Hengyang City, 20 April 2022, *M. H. Li et al. HY16383* (holotype: YUKU [YUKU02074893]!; isotypes: YUKU [YUKU02074894, YUKU02074895, YUKU02074896]!.)

#### Diagnosis.

*Rubussemiplenus* is most closely related to *Rubushirsutus* Thunb., but clearly distinguished morphologically from the latter by its habit herbs or dwarf subshrubs (vs. shrubs in *R.hirsutus*), stems villous hairs (vs. soft and glandular hairs), leaves simple (vs. imparipinnate, 3–5 foliolate), suborbicular (vs. ovate or broadly ovate), sepals ovate-orbicular (vs. ovate-lanceolate or triangular-lanceolate), petals dicyclic, semi-doubled flowers, with 10–12 (vs. petals 5).

**Figure 1.**
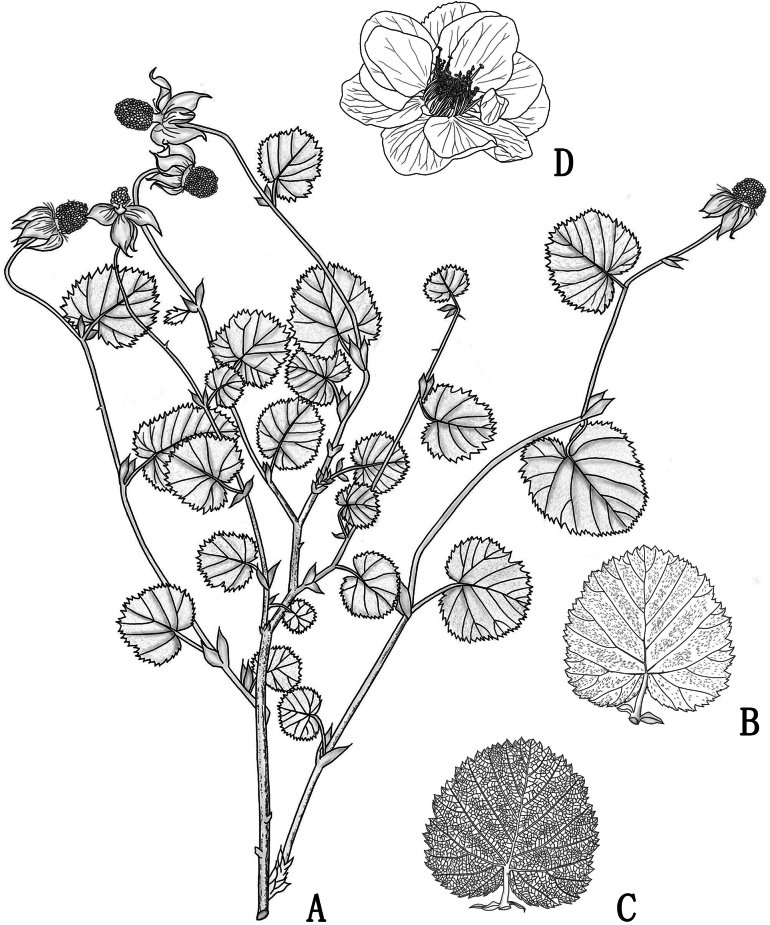
*Rubussemiplenus* sp. nov. **A** habit **B** adaxial surface of leaf **C** abaxial surface of leaf **D** flower.

#### Description.

Herbs or dwarf subshrubs, 15–20 cm in height. Stem erect, terete, and branched. Branches and branchlets green, sparsely pilose and with minute prickles. Leaves simple. Stipules persistent, adnate to base of petiole, oblong-lanceolate, pilose, entire, 0.4–0.8 (–1.0) cm long, 0.1–0.3 cm wide, apex acuminate. Petiole 0.3–2.0 cm long, pilose. Leaf blade suborbicular, 0.7–2.5 cm long, 0.7–2.5 cm wide, both surfaces pilose, lateral veins 5 pairs, base cordate, margin doubly serrate, apex rounded. Inflorescences terminal, 2–3 flowers forming a corymb; bracts oblong-lanceolate, pilose, apex acuminate. Pedicel 3.0–4.5 cm long, densely pubescent. Flowers 2.0–3.5 cm in diameter; calyx light green, pubescent or tomentose abaxially, densely tomentose adaxially; sepals 5, ovate-orbicular, 0.5–1.0 (–1.2) cm long, 0.4–0.6 cm wide, entire, apex acuminate to caudate, spreading at anthesis, reflexed in fruit; petals semi-doubled, white, dicyclic, 10–12, ovate-orbicular or broadly ovate, longer than calyx, base shortly clawed; stamens straight, numerous, glabrous; filaments linear; pistils numerous, ca. 70–100, shorter than stamens; ovary villous at base. Aggregate fruit orange-red, subglobose, 0.5–0.7 cm in diameter, glabrous or sparsely pubescent.

**Figure 2.**
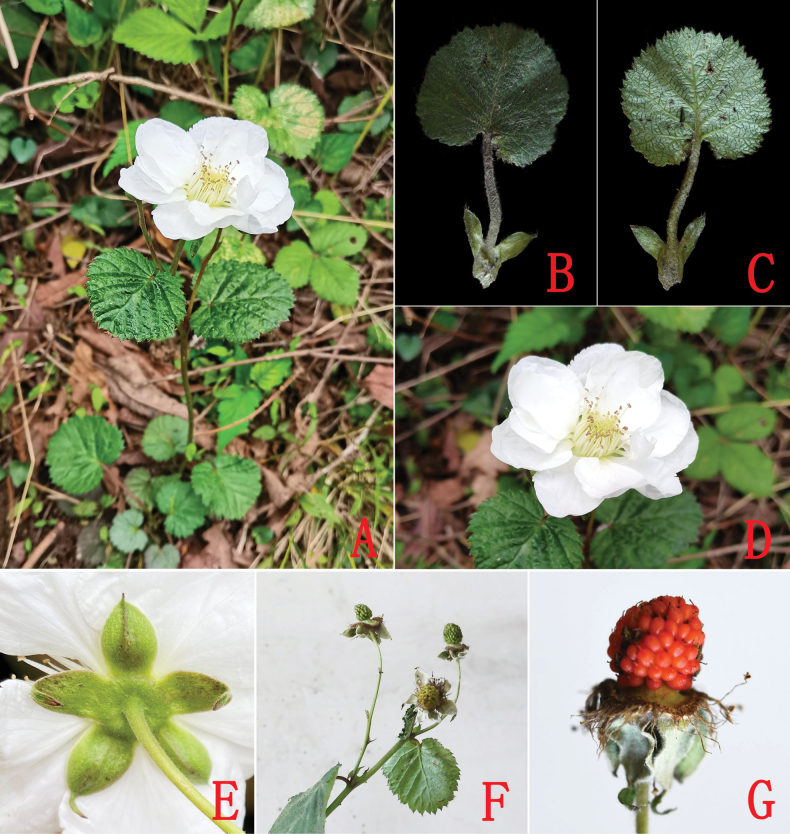
*Rubussemiplenus* sp. nov. **A** habit **B** adaxial surface of leaf **C** abaxial surface of leaf **D** flower **E** calyx **F** infructescence **G** fruit.

#### Molecular phylogenetics.

The ITS, *rbc*L, and *mat*K sequence lengths of *Rubussemiplenus* are 699 bp, 1428 bp, and 1512 bp, respectively, with GC contents of 54.1%, 43.0%, and 32.5%. Sequence alignment of the ITS dataset includes 65 sequences with 604 bp, among which 182 are parsimony-informative sites and 77 are singleton sites. The plastid (*rbc*L + *mat*K) dataset consists of 2078 aligned positions, including 125 parsimony-informative sites and 158 singleton sites. According to the Bayesian Information Criterion (BIC), the best-fit models for the ITS and *rbc*L + *mat*K datasets are TNe+R3 and K3Pu+F+R2, respectively.

**Figure 3.**
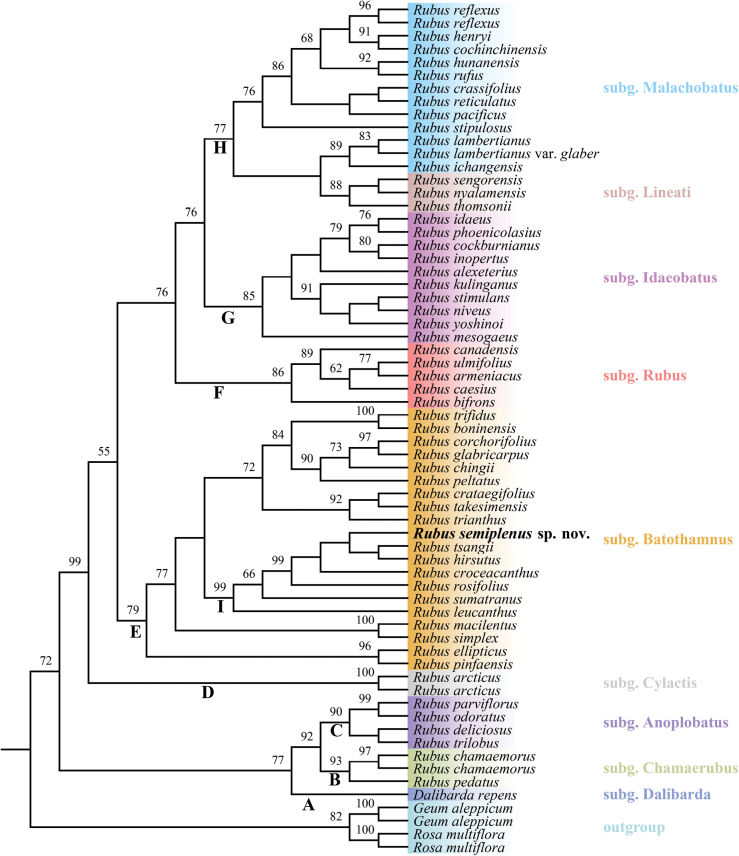
Maximum likelihood phylogenetic tree of *Rubus* based on ITS sequences, illustrating the phylogenetic placement of *R.semiplenus*. Bootstrap values are shown above branches. *R.semiplenus* is highlighted in bold face. Subgeneric names are listed on the right, with different subgenera represented by different colors. Subgeneric names and classifications follow the taxonomy proposed by Huang et al. (2023).

**Figure 4.**
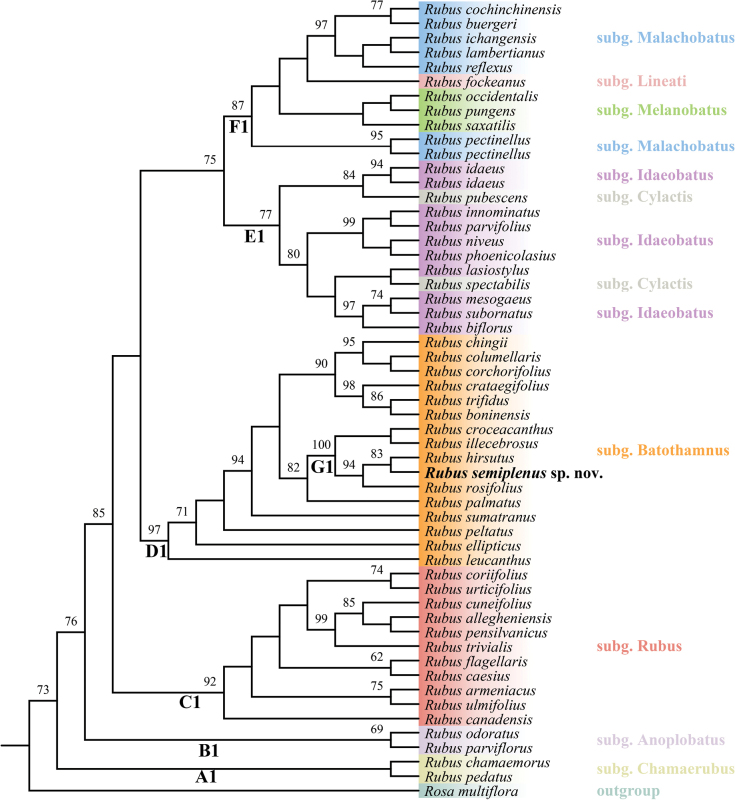
Maximum Likelihood tree of *Rubus* based on *rbc*L+*mat*K gene sequences illustrating the phylogenetic placement of *R.semiplenus*. Bootstrap values are shown above branches. *R.semiplenus* is highlighted in bold face. Subgeneric names are listed on the right, with different subgenera represented by different colors. Subgeneric names and classifications follow the taxonomy proposed by Huang et al. (2023).

The phylogenetic trees constructed using ITS and plastid (*rbc*L + *mat*K) sequences are largely congruent in topology. In the ITS phylogenetic tree (Fig. 3), the genus *Rubus* was clearly resolved into eight clades, which largely correspond to the subgenera delineated by Huang et al. (2023). *Rubussemiplenus* nests within clade E, representing subgenus Batothamnus (Focke) E. H. L. Krause, and belongs to a well-supported subclade I (BS = 99), which includes *R.tsangii* Merr., *R.hirsutus* Thunb., *R.croceacanthus* H. Lév., *R.rosifolius* Sm., *R.sumatranus* Miq and *R.leucanthus* Hance. Notably, except for *R.leucanthus*, the remaining five species in this subclade belong to the Sect. Rosaefolii as defined by Focke (1910–1914). Similar to the ITS phylogenetic tree results, in the *rbc*L + *mat*K phylogenetic tree (Fig. 4), *R.semiplenus* falls within clade D1, also representing the subgenus Batothamnus, and clusters with *R.hirsutus*, *R.rosifolius*, *R.illecebrosus* Focke, *R.croceacanthu*s to form subclade G1 with full support (BS = 100). All species in subclade G1 belong to Focke’s Sect. Rosaefolii. Additionally, *R.semiplenus* is resolved as sister to *R.hirsutus* with moderate support (BS = 83).

#### Phenology.

Flowering from March to April, Fruiting from May to June.

#### Etymology.

The specific epithet “*semiplenus*” is derived from the Greek words “*semi*” (meaning half) and “*plenus*” (meaning double), which refer to this new species with semi-double flowers.

#### Habitat and distribution.

*Rubussemiplenus* is known only from the type locality in Nanyue Hengshan National Nature Reserve, Hengyang City, Hunan Province, China. This nature reserve belongs to a subtropical monsoon humid climate and includes many Chinese National Key Protected Plants, such as *Ginkgobiloba* L., Taxuswallichianavar.mairei (Lemée & H. Lév.) L. K. Fu & Nan Li, and *Bretschneiderasinensis* Hemsl. The new species mainly grows under the shrubs at an altitude of 800–900 meters.

## ﻿Discussion

The trait of doubled or semi-doubled flowers is highly valued in horticulture for its aesthetic appeal and ornamental potential, yet it remains exceptionally rare in wild plants. Within the Rosaceae, this trait is commonly observed in many cultivated species across several genera, including *Prunus* L. and *Rosa* L. (Yang et al. 2024). However, it is exceedingly rare in the genus *Rubus*. Prior to this study, only a few species, such as Rubusrosifoliusvar.coronarius (Sims) Focke, along with a limited number of cultivated varieties, were known to exhibit this characteristic (Mao et al. 2012). Given its rarity, the discovery of *Rubussemiplenus* is of significant botanical and horticultural value.

Compared to the typical shrub habit, species with subshrub or herbaceous habits are relatively rare in genus *Rubus* (Yu and Lu 1985). However, the emergence of subshrub habits may be the result of convergent evolution, as these species often appear in different evolutionary lineages. In China, about 40 species of *Rubus* have been recorded as subshrubs or herbaceous types (Yu and Lu 1985; Lu and Boufford 2003). According to the latest subgeneric classification by Huang et al. (2023), most of the subshrub or herbaceous species belong to the Subg. Lineati (Focke) T.R.Huang & X.Y.Zhu, such as *R.fockeanus* Kurz and *R.metoensis* T. T. Yu & L. T. Lu. Species within this subgenus are mostly adapted to high altitude or high latitude habitats. Morphologically, the new species should be classified into Subg. Batothamnus (Focke) E.H.L.Krause (Huang et al. 2023). Within Subg. Batothamnus, there are also some subshrub-type *Rubus* species, such as *R.delavayi* Franch., *R.impressinervus* F. P. Metcalf and *R.simplex* Focke. Nevertheless these taxa belong to different sections and have significant taxonomic differences from the new species. Molecular analyses demonstrate that although the new species clusters within the same major clade as *R.leucanthus* Hance and *R.simplex* Focke (Figs 3, 4; Clade E, Clade D1), marked discontinuities in both phylogenetic relationships and morphological characteristics are observed.

Phylogenetic analyses based on ITS and *rbc*L + *mat*K sequences consistently indicate that the new species belongs to Rubussubg.Batothamnus (Focke) E.H.L. Krause. Moreover, *R.semiplenus* should be classified within Focke’s Sect. Rosaefolii. This result is not only supported by molecular results, but also aligns with its morphological characteristics, which are typical of species in this section, such as numerous carpels and white flowers. However, all previously known species in this section are shrubs with compound leaves. In contrast, *R.semiplenus* is unique, being the only species in this section with simple leaves and semi-double flowers, further distinguishing it from other members of the group.

Molecular analyses indicate that the new species is most closely related to *R.hirsutus*. However, it can be readily distinguished from *R.hirsutus* by several characteristics. The new species is a herb or dwarf subshrub, whereas *R.hirsutus* is a shrub. Its stems are villous, compared to the soft and glandular hairs of *R.hirsutus*. The leaves are simple, whereas *R.hirsutus* has imparipinnate leaves with 3–5 leaflets. The leaf blades are suborbicular, whereas those of *R.hirsutus* are ovate to broadly ovate. The petals are arranged in two cycles, with 10–12 petals, in contrast to the five petals of *R.hirsutus*. Additionally, the fruits are glabrous or sparsely pubescent, whereas those of *R.hirsutus* are entirely glabrous.

### ﻿Additional specimens examined

*Rubushirsutus*. CHINA. Jiangxi: Fengxin County, 11 May 1963, Liu SL et al. 1169 (IBSC-0324075). Jiangsu: Nanjing City, June 1955, Guan KJ 147 (HENU-0180581). Anhui: Taiping County, at 700 m, June 1959, Herbarium of the Institute of Botany 2056 (PE-00250149). Fujian: Wuyishan City, Mount Wuyi, at 170 m, 4 April 1955, Wang MJ et al. 3003 (NAS-00365571). Henan: Xinyang City, kikungshan, at 700 m, 12 May 1998, Yang H 97053153 (HENU-1016206). Zhejiang: Hangzhou City, Changhua Town, at 650 m, 9 May 1957, He XY 33648 (IBSC-0324088).

## Supplementary Material

XML Treatment for Rubus semiplenus

## ﻿Additional information

### Author ORCIDs

Ting-Ting Wang https://orcid.org/0009-0000-1593-1738

Huan-Chong Wang https://orcid.org/0000-0001-8562-8849

### Data availability

All of the data that support the findings of this study are available in the main text.

## ﻿Appendix 1

**Table A1.** ITS sequences information downloaded from the GenBank.

No.	Species	GenBank
1	*Dalibardarepens* L.	AF055748
2	*Geumaleppicum* Jacq.	KF873777
3	*Geumaleppicum* Jacq.	OR699355
4	*Rosamultiflora* Thunb.	KP966598
5	*Rosamultiflora* Thunb.	MK533408
6	*Rubusalexeterius* Focke	EF034119
7	*Rubusarcticus* L.	AF055741
8	*Rubusarcticus* L.	EF034120
9	*Rubusarmeniacus* Focke	OL899237
10	*Rubusbifrons* Vest	KM037669
11	*Rubusboninensis* Koidz.	LC624385
12	*Rubuscaesius* L.	KU294729
13	*Rubuscanadensis* L.	AF055777
14	*Rubuschamaemorus* L.	AF055740
15	*Rubuschamaemorus* L.	U90803
16	*Rubuschingii* Hu	LC624375
17	*Rubuscochinchinensis* Tratt.	OP340989
18	*Rubuscockburnianus* Hemsl.	KU881070
19	*Rubuscorchorifolius* L.	KP093158
20	*Rubuscrassifolius* T. T. Yu & L. T. Lu	KU881076
21	*Rubuscrataegifolius* Bunge	AF055754
22	*Rubuscroceacanthus* H. Lév.	LC624288
23	*Rubusdeliciosus* Torr.	AF055733
24	*Rubusellipticus* Sm.	EF034124
25	*Rubusglabricarpus* W. C. Cheng	KU881090
26	*Rubushenryi* Hemsl. & Kuntze	KU881091
27	*Rubushirsutus* Thunb.	LC515691
28	*Rubushunanensis* Hand.-Mazz.	KU881093
29	*Rubusichangensis* Hemsl. & Kuntze	EF034128
30	*Rubusidaeus* L.	AF055755
31	*Rubusinopertus* (Focke) Focke	KU881107
32	*Rubuskulinganus* L. H. Bailey	KU881111
33	*Rubuslambertianus* Ser.	AF055796
34	Rubuslambertianusvar.glaber Hemsl.	KU881114
35	*Rubusleucanthus* Hance	KP093160
36	*Rubusmacilentus* Cambess.	EF034136
37	*Rubusmesogaeus* Focke ex Diels	LC515690
38	*Rubusniveus* Wall. ex G. Don	MZ206307
39	*Rubusnyalamensis* T. T. Yu & L. T. Lu	KU881127
40	*Rubusodoratus* L.	KM037685
41	*Rubuspacificu*s Hance	KU881128
42	*Rubusparviflorus* Nutt.	EF050804
43	*Rubuspedatus* Sm.	LC624387
44	*Rubuspeltatus* Maxim.	LC515683
45	*Rubusphoenicolasius* Maxim.	AF055759
46	*Rubuspinfaensis* H. Lév. & Vaniot	KU881148
47	*Rubusreflexus* Ker Gawl.	KP093165
48	*Rubusreflexus* Ker Gawl.	KP093166
49	*Rubusreticulatus* Wall. ex Hook. f.	KU881158
50	*Rubusrosifolius* Sm.	KP093168
51	*Rubusrufus* Focke	KU881162
52	*Rubussengorensis* Grierson & D.G.Long	EF034141
53	*Rubussemiplenus* Huan C. Wang, Ming Hong Li & T. T. Wang	PV596324
54	*Rubussimplex* Focke	KU881169
55	*Rubusstimulans* Focke	KU881172
56	*Rubusstipulosus* T. T. Yu & L. T. Lu	KU881173
57	*Rubussumatranus* Miq.	LC515688
58	*Rubustakesimensis* Nakai	AY818216
59	*Rubusthomsonii* Focke	EF034145
60	*Rubustrianthus* Focke	AY083366
61	*Rubustrifidus* Steud.	AF055737
62	*Rubustrilobus* Ser.	AF055738
63	*Rubustsangii* Merr.	KU881190
64	*Rubusulmifolius* Schott	KM037610
65	*Rubusyoshino*i Koidz.	LC624345

**Table A2.** *rbc*L, *mat*K gene sequences information downloaded from the GenBank.

No.	Species	*rbc*L	*mat*K
1	*Rosamultiflora* Thunb.	LC662004	LC662012
2	*Rubusallegheniensis* Porter	MK526532	MK520547
3	*Rubusarmeniacus* Focke	KX678957	KX677516
4	*Rubusbiflorus* Buch.-Ham. ex Sm.	MN192849	MN492833
5	*Rubusboninensis* Koidz.	LC624541	LC624475
6	*Rubusbuergeri* Miq.	LC624530	LC624464
7	*Rubuscaesius* L.	JN892997	JN895641
8	*Rubuscanadensis* L.	KY427303	KY427300
9	*Rubuschamaemorus* L.	LC624523	LC624457
10	*Rubuschingii* Hu	LC624531	LC624465
11	*Rubuscochinchinensis* Tratt.	GU363824	GU363786
12	*Rubuscolumellaris* Tutcher	KP094935	KP093990
13	*Rubuscorchorifolius* L.	LC797503	LC797488
14	*Rubuscoriifolius* Liebm.	OP711219	OQ289878
15	*Rubuscrataegifolius* Bunge	MZ128512	MZ128648
16	*Rubuscroceacanthus* H. Lév.	LC515896	LC515924
17	*Rubuscuneifolius* Pursh	ON341340	ON341187
18	*Rubusellipticus* Sm.	MK157064	MK157063
19	*Rubusflagellaris* Willd.	KP643972	KP643060
20	*Rubusfockeanus* Kurz	MH116377	MH116826
21	*Rubushirsutus* Thunb.	LC629708	LC629702
22	*Rubusichangensis* Hemsl. & Kuntze	MN192853	MN492835
23	*Rubusidaeus* L.	LC624542	LC624476
24	*Rubusidaeus* L.	LC629709	LC629703
25	*Rubusillecebrosus* Focke	MZ128496	MZ128630
26	*Rubusinnominatus* S. Moore	MN205247	MN492837
27	*Rubuslambertianus* Ser.	LC624532	LC624466
28	*Rubuslasiostylus* Focke	MH657789	MH659334
29	*Rubusleucanthus* Hance	KP094382	KP093463
30	*Rubusmesogaeus* Focke ex Diels	MN205248	MN492838
31	*Rubusniveus* Wall. ex G. Don	MK157050	MK157049
32	*Rubusoccidentalis* H. Lév.	LC515877	LC515905
33	*Rubusodoratus* L.	MF349716	MF349835
34	*Rubuspalmatus* Hemsl.	LC624560	LC624494
35	*Rubusparviflorus* Nutt.	MK526550	MK520559
36	*Rubusparvifolius* Nutt.	MN205250	MN492841
37	*Rubuspectinellus* Maxim.	LC624529	LC624463
38	*Rubuspectinellus* Maxim.	MZ128501	MZ128636
39	*Rubuspedatus* Sm.	LC624543	LC624477
40	*Rubuspeltatus* Maxim.	MZ128447	MZ128566
41	*Rubuspensilvanicus* Poir.	KP643958	KP643050
42	*Rubusphoenicolasius* Maxim.	MZ128485	MZ128617
43	*Rubuspubescens* Raf.	MG249679	MG221118
44	*Rubuspungens* Cambess.	LC624547	LC624481
45	*Rubusreflexus* Ker Gawl.	KP094770	KP093831
46	*Rubusrosifolius* Sm.	MN192857	MN451028
47	*Rubussaxatilis* L.	MK925451	MK926261
48	*Rubussemiplenus* Huan C. Wang, Ming Hong Li & T. T. Wang	PV600993	PV600992
49	*Rubusspectabilis* Pursh	LC515898	LC515926
50	*Rubussubornatus* Focke	MH116380	MH116829
51	*Rubussumatranus* Miq.	LC515888	LC515916
52	*Rubustrifidus* Steud.	MZ128464	MZ128649
53	*Rubustrivialis* Michx.	OL434824	OL434947
54	*Rubusulmifolius* Schott	HM850315	HM850696
55	*Rubusurticifolius* Poir.	JQ593603	JQ588899
